# Supplementary material for: Omni-resonant optical micro-cavity
Source: Sci Rep. 2017 Sep 4;7:10336. doi: 10.1038/s41598-017-10429-4 (PMC5583348; doi:10.1038/s41598-017-10429-4)
Supplement: Supplementary file 1 — Supplementary Information [file 41598_2017_10429_MOESM1_ESM.pdf]

Omni-resonant optical micro-cavity

Soroush Shabahang[1], H. Esat Kondakci[1], Massimo L. Villinger[1], Joshua D. Perlstein[2], Ahmed El Halawany[1], and Ayman F. Abouraddy[1,2*]

[1]CREOL, The College of Optics & Photonics, University of Central Florida, Orlando, FL 32816, USA

[2]Materials Science and Engineering Department, College of Engineering and Computer Science, University of Central Florida, Orlando, FL 32816, USA

[*]raddy@creol.ucf.edu

Supporting Information

1. Structure of the Fabry-Pérot cavity

The planar Fabry-Pérot (FP) cavity used in the main text is composed of two symmetric 5 bilayer Bragg mirrors enclosing a 4-μm-thick SiO_2 dielectric spacer (on a BK7 substrate). The overall structure thus has the layered form:

Incidence → Air – $(HL)_5$ – SiO_2 – $(LH)_5$ – BK7.

Here each bilayer (HL) consists of a high-index (H) and low-index (L) material, which are TiO_2 and SiO_2, respectively. The measured refractive indices for TiO_2 and SiO_2 at representative wavelengths of interest are given in Table S1 and Table S2 (provided by Blue Ridge Optics, LLC). The TiO_2 films were formed by evaporating Ti_2O_3 source material under O_2 partial pressure. Using these values in Tables S1 and S2, we calculated the spectral transmission of the mirror and cavity at normal incidence, Fig. S1 and Fig. S2, respectively, and the spectral-angular response of the cavity (Fig. S3). The spectral simulations employ the transfer matrix method at a resolution of (π/2)/500.

Table S1. Refractive index of TiO_2 (produced from Ti_2O_3)

λ	n
470	2.1
510	2.09
550	2.085
590	2.08
670	2.075

Table S2. Refractive index of SiO_2

λ	n
450	1.4793
500	1.4780
550	1.4772
570	1.4769
600	1.4766

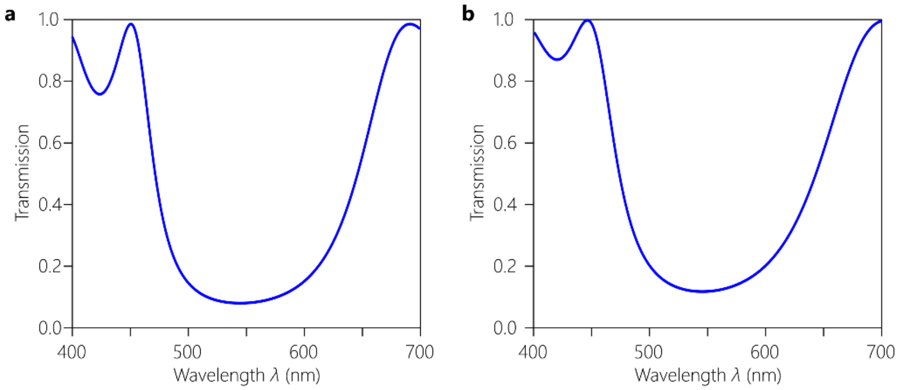

Figure S1 | Spectral transmission through the 5-bilayer Bragg mirror at normal incidence. **a**, The mirror is surrounded by glass on one side and air from the other. **b**, Mirror is surrounded by glass on both sides.

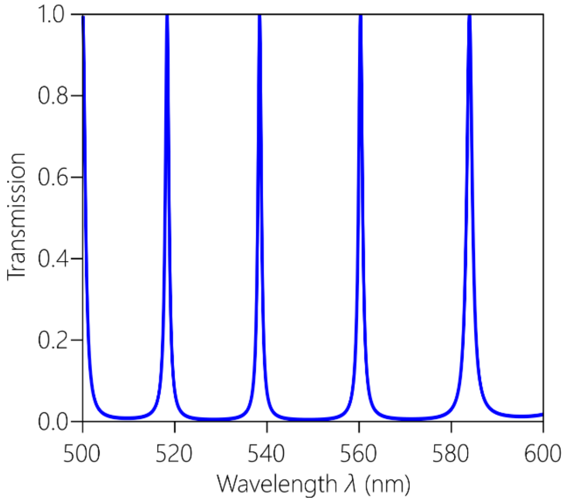

Figure S2 | Spectral transmission through the FP cavity at normal incidence. The free spectral range is ~ 25 nm.

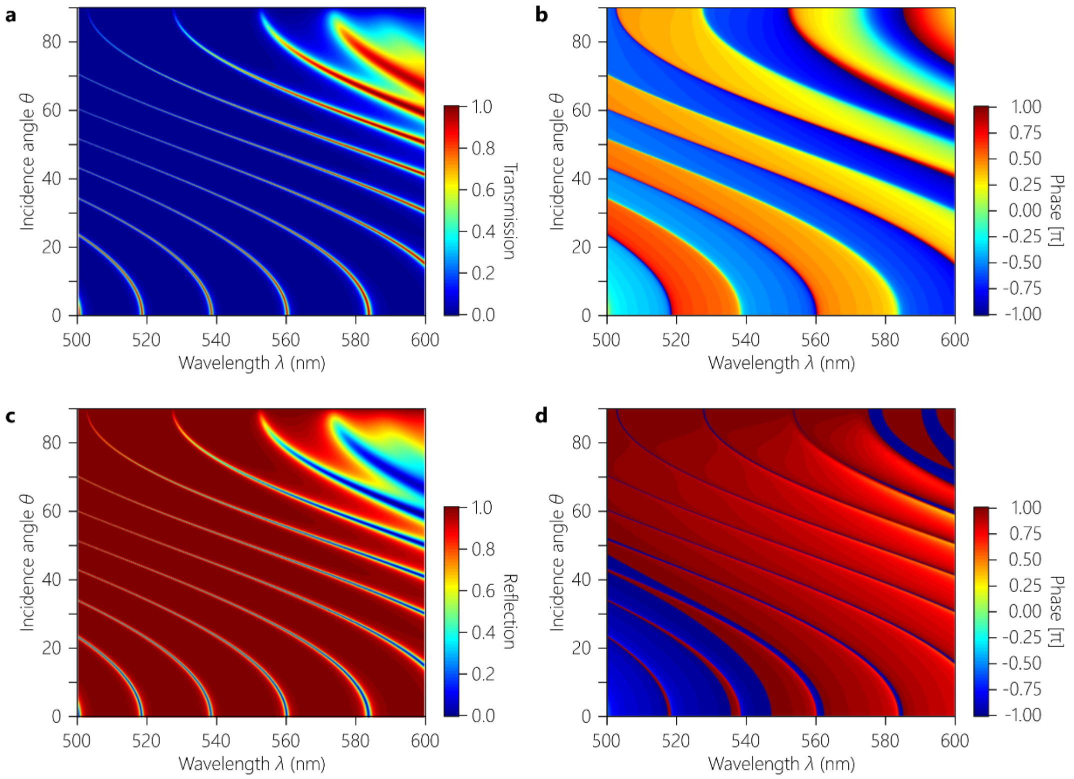

Figure S3 | Spectral-angular transmission through and reflection from the FP cavity for incidence of the TE polarization from air. a,**b**, (**a**) Transmittance amplitude and (**b**) phase as a function of the angle of incidence in the spectral range of the Bragg mirror bandgap; see Fig. S1. **c**,**d**, (**c**) Reflectance amplitude and (**d**) phase. Compare (**a**) to the experimental results reported in Fig. 3a of the main text.

S2. Simulation of the achromatic resonances

In this Section, we calculate the transmission characteristics of the FP cavity when it is inserted into a setup that induces achromatic resonances. In particular, we simulate the effect of the grating and lens L_1 placed in the path of a collimated broadband beam, as shown in Fig. 3b of the main text.

We assume an ideal grating with TE or TM polarized collimated light directed at an incidence angle $\alpha = 50°$ with respect to the normal to the grating. See Fig. S4 for a schematic of the setup that highlights the definition of the relevant angles for our analysis. The angularly dispersed light from the grating is then directed to the sample through the lens L_1. We assume that 550 nm is the central wavelength and take it to define the optical axis. The tilt angle of the sample ψ is measured with respect to this optical axis. We define the angle $\gamma(\lambda)$, which is the diffraction angle with respect to the grating normal. The central wavelength $\lambda_c = 550$ nm is diffracted at $\gamma_o = \gamma(\lambda_c = 550 \text{ nm})$ and coincides with the optical axis. The angle any wavelength λ makes with respect to this optical axis is $\gamma(\lambda) - \gamma_o$. This angle is boosted via the lens L_1 by a ratio $\frac{d_1}{d_2}$, where d_1 and d_2 are the distances from the grating to L_1 and from L_1 to the cavity, respectively. The incidence angle made by a wavelength λ after the lens with respect to the optical axis is thus:

$$\varphi(\lambda) = \tan^{-1}\left\{\frac{d_1}{d_2}\tan(\gamma(\lambda) - \gamma_o)\right\},$$

with $\varphi_o = \varphi(\lambda_c = 550 \text{ nm}) = 0$. The distances d_1 and d_2 are selected such that the illuminated spot on the grating is imaged onto the cavity. If the focal length of L_1 is f, then $d_2 = \frac{f d_1}{f - d_1}$. When the cavity is oriented such that it is perpendicular to the optical axis, the angle of incidence of each wavelength is $\varphi(\lambda)$. Upon tilting the cavity by ψ, the angle of incidence with respect to the normal to the cavity is $\theta(\lambda) = \varphi(\lambda) + \psi$.

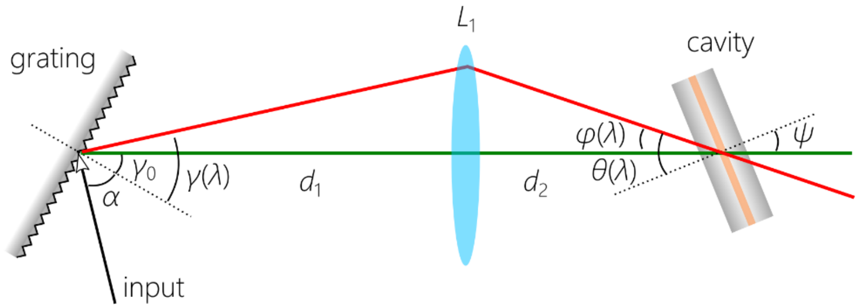

Figure S4 | Schematic of the configuration of the grating and cavity to highlight the definitions of the various relevant angles. α and $\gamma(\lambda)$ are measured with respect to the normal to the grating. The optical axis (shown in green) coincides with $\gamma_o = \gamma(\lambda_c = 550 \text{ nm})$. $\varphi(\lambda)$ is measured with respect to the optical axis, while $\theta(\lambda)$ is measured from the normal to the cavity: $\theta(\lambda) = \varphi(\lambda) + \psi$, where ψ is the tilt angle of the cavity with respect to the optical axis.

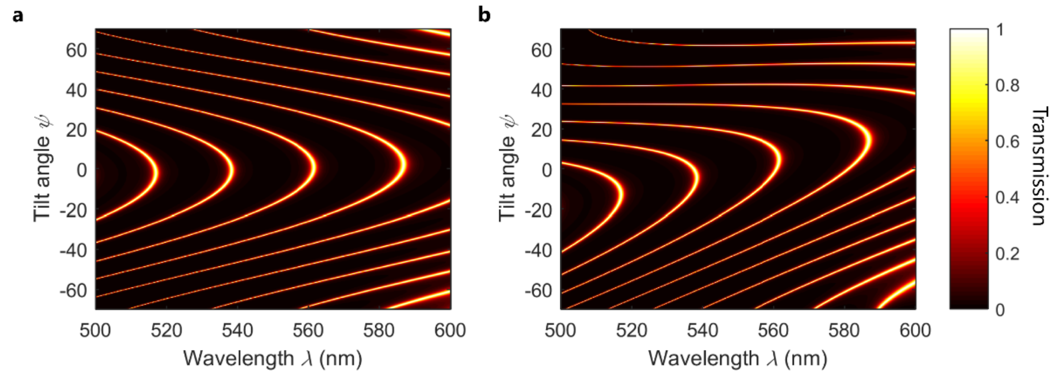

Figure S5 | Spectral transmission through an achromatic resonator for TE polarization while varying the cavity tilt angle ψ. a, The focal length of L_1 is $f = 50$ mm and $d_2 = 8$ cm. **b**, Same as (**a**) for $f = 25$ mm and $d_2 = 12$ cm. Compare (**a**) and (**b**) to the measurements in Fig. 3c and Fig. 3d in the main text.

With these parameters, we calculate the transmission through the sample using the transfer matrix method for both TE (Fig. S5) and TM (Fig. S6) polarizations. We carry out the calculations for two values of the focal length, $f = 50$ mm (Fig. S5a) and $f = 25$ mm (Fig. S5b) corresponding to the values used in our experiment. The calculations in Fig. S6a and Fig. S6b are to be compared to the measurements reported in the main text in Fig. 3c and Fig. 3d, respectively.

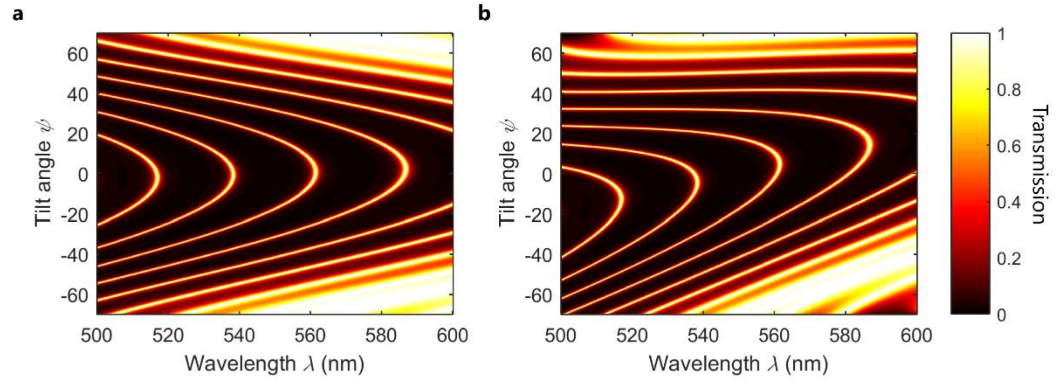

Figure S6 | Same as Fig. S5 for TM polarization.

S3. Experimental setup

Details of the experimental setup for measuring spectral transmission

In the experimental setup shown schematically in Fig. 3b in the main text, white light emitted from a halogen source (Thorlabs, QTH10/M) is spatially filtered by coupling through a 1-m-long multimode fiber (50-μm-diameter) and then collimated via a fiber collimator, followed by a polarizer to control the state of polarization of the beam. A grating (Thorlabs GR25-1850) is placed at ~ 30 cm from the collimator and orientated at 50° with respect to the incident light. Before incidence on the grating, the

beam passes through a 1-mm-wide vertical slit. Diffracted light is focused by a lens L_1 on the cavity, which is mounted on a rotational stage. The lens is placed 12 cm away from the grating to provide the appropriate angular dispersion for phase-matching of the wave-vector axial component. Light transmitted though the cavity is collimated by a second 25-mm-focal-length lens L_2 and then coupled into a multimode fiber using an aspherical lens (15-mm-focal-length) connected to a spectrometer (JAZ, Ocean Optics). The wavelength resolution of the measurement is limited by the multimode fiber to ~ 1 nm.

Light diffracted by the grating is spread horizontally, so transversal displacements of optical components can spectrally shift the resonances. To align the setup for the desired spectral range, care must be taken to ensure that the center wavelength of $\lambda_c = 550$ nm passes through the center of the lenses L_1 and L_2 and thus defines the optical axis. To maximize the achromatic resonance bandwidth, the focusing lens L_1 is first placed in the desired distance from the grating obtained from geometrical optics considerations. The collection aspherical lens and fiber are then aligned to collect the maximum spectral bandwidth. The cavity is then mounted on the rotational stage at the focal point of the lens L_1. Although axial displacement of the resonator does not affect the resonances, it can alter the angular distribution of the beam after L_1, a feature we use to fine-tune the bandwidth of the resonances.

Details of the experimental setup for imaging through the cavity

The cavity appears like a mirror at near normal incidence (Fig. 3b inset in the main text), however it transmits most of the incident light in the achromatic resonance configuration and thus appears transparent. To visually demonstrate the cavity transparent, an object (an opaque letter ‘i’ on a transparent substrate) is imaged onto a CCD camera through the cavity. We first imaged the object through the cavity alone (Fig. 4c in the main text). The cavity is oriented normally with respect to the beam path and as a result blocks most of the optical power due to very low optical throughput and instead reflects the incident beam (except on resonance). The setup is sketched in Fig. S7.

We next carry out the measurement in the achromatic resonance configuration; see Fig. S8. An H-polarized (TE) collimated white light beam is incident on the object (letter ‘i’ on a transparent substrate), is angularly dispersed by the reflective diffraction grating, followed by passage through the lens L_1 with focal length $f = 25$ mm. The beam passes through the cavity mounted on a rotation stage and is collimated by a second 25-mm-focal-length lens L_2. A second reflective grating restores the original beam structure and finally a 10-cm-focal lens L_3 images the object onto a CCD camera (Imaging Source, DFK 31BU03). The distances from L_3 to the grating and from L_3 to the CCD are both ~ 10 cm. As a reference, we carry out the experiment using this setup but in absence of the cavity (Fig. 4b in the main text). The results of the imaging experiment in presence of the cavity are presented in Fig. 4d of the main text. At certain values of ψ, the resonance broadens, and when the achromatic resonance condition is met the optical throughput increases.

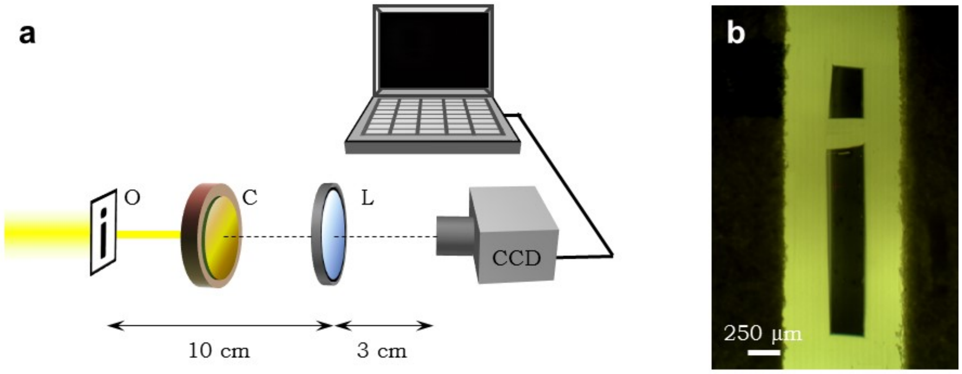

Figure S7 | Imaging through an achromatic resonance. a, Optical setup for imaging an object (letter 'i') through the FP cavity. L: imaging lens; C: FP cavity; O: object plane. **b**, Optical transmission micrograph of the object.

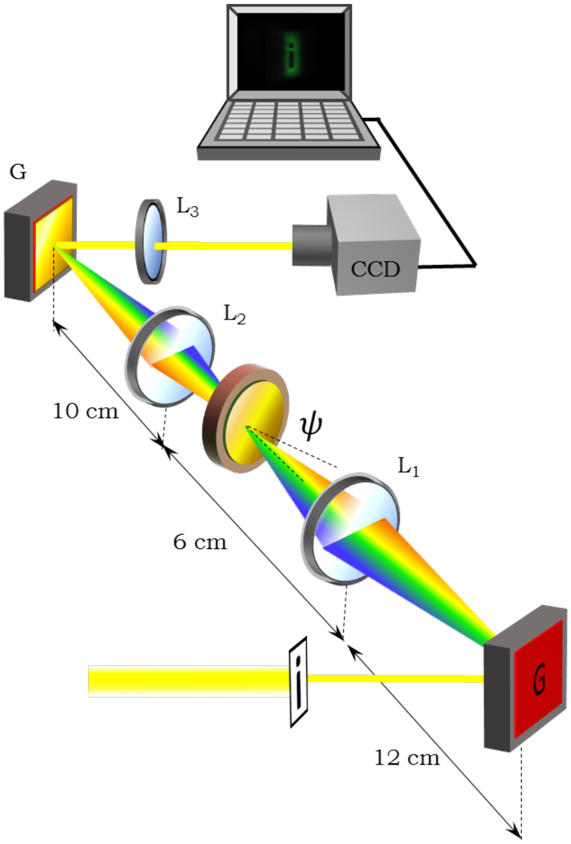

Figure S8 | Optical setup for imaging an object (letter 'i') through the FP cavity in a configuration that induces achromatic transmission. G: grating; L_1, L_2, and L_3: lenses.
